# Focusing on life rather than illness: the lived experience of children with life-threatening and life-limiting conditions—a qualitative study

**DOI:** 10.1177/26323524241301431

**Published:** 2024-11-29

**Authors:** Trine Brun Kittelsen, Charlotte Castor, Anja Lee, Lisbeth Gravdal Kvarme, Anette Winger

**Affiliations:** Department of Nursing and Health Promotion, Faculty of Health Sciences, Oslo Metropolitan University, Pilestredet 32, 0167 Oslo, Norway; Department of Health Sciences, Lund University, Lund, Sweden; Division of Pediatric and Adolescent Medicine, Oslo University Hospital HF, Oslo, Norway; Department of Nursing and Health Promotion, Faculty of Health Sciences, Oslo Metropolitan University, Oslo, Norway; Department of Nursing and Health Promotion, Faculty of Health Sciences, Oslo Metropolitan University, Oslo, Norway

**Keywords:** children, life-limiting condition, life-threatening condition, lived experience, meaningful activity, participation, pediatric palliative care

## Abstract

**Background:**

The perspectives of children with life-threatening or life-limiting (LT/LL) conditions have predominantly been conveyed through their parents rather than heard from the children themselves. Despite an increase in studies focusing on children’s perspectives in pediatric palliative care, this research remains limited, particularly in including children who are unable to express themselves orally.

**Objective:**

This study seeks to address gaps in existing knowledge, especially the limited inclusion of children’s perspectives and the exclusion of children with communicative and cognitive disabilities. The aim of this study was to explore the lived experiences of children living with LT/LL conditions.

**Design:**

The study has a qualitative, hermeneutic phenomenological design inspired by van Manen’s phenomenology of practice.

**Methods:**

Twelve children with cancer or genetic conditions participated. Due to the children’s varying cognitive and communicative abilities, multiple data collection methods were employed to capture the children’s perspectives, including interviews, interactions, descriptions of the children’s non-verbal expressions, and an adapted photo elicitation method. Data were analyzed using thematic analysis.

**Results:**

The findings showed that the children’s attention revolved around life rather than illness. The analysis revealed the presence of three themes: wanting to engage in life, being dependent on familiar relations, and the importance of cherished items.

**Conclusion:**

Professionals across various levels within pediatric palliative care should acknowledge children’s desire to engage in life despite serious illness and facilitate participation. Practices should be implemented to support children’s agencies and expressions of what is important to them. This recognition can guide care plans and interventions at all levels of pediatric palliative care when a child is living with an LT/LL condition. The study emphasizes that children with LT/LL conditions are children first and foremost, with a fundamental need to participate in meaningful activity, just like any other child.

## Introduction

Globally, approximately 21 million children (aged 0–18 years) live with life-threatening or life-limiting (LT/LL) conditions.^1^ The number of children in need of pediatric palliative care (PPC) in Norway is not known, but estimates based on recent studies from the United Kingdom indicate that approximately 8000 children are living with LT/LL conditions in Norway.^2^ According to the World Health Organization, all children with LT/LL conditions have the right to receive PPC, regardless of their diagnosis or stage of illness.^3^ PPC seeks to enhance the overall quality of life (QoL) for both the child and the family by addressing and alleviating physical, psychological, social, and spiritual suffering.^3^ The spectrum of diagnoses within PPC is wide and heterogeneous,^4^ with non-cancer conditions accounting for the majority of children with LT/LL.^4,5^ The prolonged duration of many conditions necessitates PPC for varying durations, spanning from days to months or even years.^6,7^

Systematic reviews indicate that the perspectives of ill children are predominantly obtained from their parents rather than directly from the children themselves.^8,9^ However, in some studies, children with LT/LL conditions have provided their perspectives directly. In these studies, they have described a loss of normality,^10,11^ changes in relationships within families and social circles,^10–13^ physical concerns,^11–13^ and the condition’s impact on their ability to engage in age-appropriate activities.^11,12^ Other studies have compared how parents and ill children themselves report the child’s symptom burden and QoL. In these studies, children tend to report lower symptom burden^14^ and higher QoL than their parents’ reports.^15,16^ This shows that by including children in research, new insights into their worlds can arise, as children’s perspectives may not fit with adult assumptions about their concerns.^17^

According to the United Nations Convention on the Rights of the Child, all children are granted legal rights to participate in activities and decisions concerning them. In the last 30 years, there has been a growing awareness about involving children in research.^18–20^ Nevertheless, within PPC, a large group of children with communicative, cognitive, and motor disabilities are often excluded from research.^21–23^ Children with reduced ability to orally express themselves are often unable to give informed consent,^24^ and they may need other ways to be heard than those who are able to communicate orally. These limitations are not only due to the children’s reduced verbal communication skills but also due to the lack of experience, time, or commitment from others to understand how children with limited abilities express themselves.^21^ Fundamental to achieving the overall aim of participation is to use flexible research designs^25^ and methods that can maximize children’s potential to express themselves,^21,26^ rather than focusing on what children with complex developmental disabilities do not have the capacity to do.^27^ Methods such as participant observation,^28^ seeking intimate information through familiar persons on how the child interacts and responds to the world,^17^ and gaining a deep understanding of the context in which the children live^29^ may facilitate an understanding of the child’s experiences that cannot be achieved through other methods. However, all of these methods require careful consideration and reflexivity regarding how data are collected and analyzed, how they are interpreted, and how the children’s contributions are represented in the findings.^17,19,30–33^

As the experience of the child is crucial when planning for clinical practice, children should be included in research regardless of their ability to communicate verbally. It is recommended to use qualitative approaches to provide detailed information on the child’s subjectivity.^9^ Following these recommendations, this study aimed to explore the lived experiences of children with LT/LL conditions using a qualitative approach.

## Methods

### Design

The study has an inductive qualitative design, using a hermeneutic phenomenological approach inspired by van Manen’s phenomenology of practice.^34^ Phenomenology of practice is a context-sensitive form of descriptive and interpretive inquiry in which an understanding of the phenomenon can lead to more sensitive and caring professional services.^35^ Lived experiences are concrete and pre-reflected experiences that give us access to understanding the phenomenon under study,^35^ which in this case is the phenomenon of children living with an LT/LL condition. The research process was guided by van Manen’s lifeworld existentials—lived time, lived body, lived space, and lived relation—which are described as the fundamental structure of every person’s lifeworld.^35^ A key distinction in research with children is the difference between the *child’s perspective* (the child’s own view in a situation) and the *child perspective* (adults taking the perspective of the child).^30^ In this study, the aim was to capture the child’s perspective through sensitivity to the child’s own expressions.^36,37^ The reporting of this study conforms to the consolidated criteria for reporting qualitative research (COREQ; Supplemental Material).^38^

### Recruitment and participants

The current study was part of a family study with three substudies on the ill child’s, the sibling’s,^39^ and the parents’^40^ experiences of living with an LT/LL condition. For this article, the aim was to explore ill children’s lived experiences. We employed two methods for recruiting families: through three hospitals in the southeastern health region of Norway, and by engaging with three user organizations in the Children in Palliative Care research network.^41^ Contact persons in the hospitals facilitated family recruitment by directly approaching parents and inquiring whether they wished to participate in the study. The user organizations disseminated information about the study to parents through meetings, a closed Facebook group, and a webpage. Families expressing interest in participation granted consent to be contacted by the first author, except for two families who initiated direct contact with the first author independently. One family opted not to participate after receiving more information. We included children younger than 18 years in the phase of ongoing care, regardless of the child’s LT/LL diagnosis, cognitive functioning, or the children’s different ways of expressing themselves. We adopted an inclusive recruitment strategy, refraining from excluding children, and instead aimed to understand each child’s unique circumstances.

We recruited a total of 12 children and their families (Table 1) from September 2021 to March 2022. The authors did not have any prior knowledge of the children or their families. In two families, the ill child was the only child. All children resided with both their mother and father. Three children were diagnosed with cancer, and nine children had genetic conditions. The children with genetic conditions had extensive medical complexity, with various grades of permanent cognitive and motor impairment, and additional conditions such as epilepsy and visual impairment. Four of the children communicated verbally, and eight communicated with other augmentative or alternative communication forms (AACs; Table 2). AAC refers to any method used to communicate other than speech^42^ and can include aided AAC, such as equipment or assistive technology, or unaided/body-based AAC, such as vocalizations, body language, facial expressions, or signs.^43^ All participants spoke Norwegian.

**Table 1.** Demographic characteristics of the ill children (*n* = 12), their parents (*n* = 24), and their siblings (*n* = 13^a^).

Characteristics	*n*
Ill child age (years)	1–17 (median 5)
Ill child gender	
Female	5
Male	7
Parents’ age	
Mothers	27–53 (median 38)
Fathers	32–58 (median 40)
Parents’ education level	
Upper secondary school	11
Bachelor’s degree	8
Graduate studies	5
Sibling age	3–29 (median 10)
Sibling gender	
Female	6
Male	7

a Seven siblings provided descriptions of the ill child’s non-verbal expressions in concrete situations.

**Table 2.** Data collection methods used for each child.

Child	Data collection method	The ill child’s communication form^a^
Child 1 Age 17	Interaction with the ill child Parents’ and two siblings’ descriptions of concrete situations where the ill child’s perspective was revealed through non-verbal expressions Object and picture	Verbal, with limited speech*Unaided AAC* Body language *Aided AAC* Electronic AAC device for a simple conversation about known themes
Child 2 Age 5	Interaction with the ill child Parents’ and one sibling’s descriptions of concrete situations where the ill child’s perspective was revealed through non-verbal expressions Object and picture	*Unaided AAC* Sounds and body language *Aided AAC* Symbols used to give information to the child, and electronic devices for one-sentence messages
Child 3 Age 5	Interview with the ill child Parents’ descriptions of concrete situations where the ill child’s perspective was revealed through non-verbal expressions Object and picture	Verbal
Child 4 Age 5	Interaction with the ill child Parents’ and one sibling’s descriptions of concrete situations where the ill child’s perspective was revealed through non-verbal expressions Object	*Unaided AAC* Sounds Body language
Child 5 Age 4	Interview with the ill child Parents’ and one sibling’s descriptions of concrete situations where the ill child’s perspective was revealed through non-verbal expressions Object and picture	Verbal
Child 6 Age 3	Interaction with the ill child Parents’ and two siblings’ descriptions of concrete situations where the ill child’s perspective was revealed through non-verbal expressions Object and picture	*Unaided AAC* Sounds Body language
Child 7 Age 8	Interview with the ill child Parents’ descriptions of concrete situations where the ill child’s perspective was revealed through non-verbal expressions Object	*Verbal*, with limited speech
Child 8 Age 13	Interaction with the ill child Parents’ descriptions of concrete situations where the ill child’s perspective was revealed through non-verbal expressions Pictures	*Unaided AAC* Sounds Facial expressions
Child 9 Age 5	Interaction with the ill child Parents’ descriptions of concrete situations where the ill child’s perspective was revealed through non-verbal expressions	*Unaided AAC* Sounds Facial expressions Body language
Child 10 Age 7	Interview with the ill child Parents’ descriptions of concrete situations where the ill child’s perspective was revealed through non-verbal expressions Object and picture	Verbal
Child 11 Age 17	Interaction with the ill child Parents’ descriptions of concrete situations where the ill child’s perspective was revealed through non-verbal expressions Objects	*Verbal with limited speech* *Unaided AAC* Body language
Child 12 Age 1	Interaction with the ill child Parents’ descriptions of concrete situations where the ill child’s perspective was revealed through non-verbal expressions	*Unaided AAC* Facial expression Body language

a Verbal, aided or unaided augmentative or alternative communication form (AAC).

### Data collection

A pilot interview was conducted with one family comprising two parents, a child with an LL condition aged 15, and three siblings aged 5, 10, and 12 (not included in the material). The interview provided insights into the challenges of addressing research questions with a child who has cognitive and communicative disabilities, as well as the importance of involving both siblings and parents in providing concrete descriptions of the ill child’s non-verbal expressions.

All data collection was conducted by TBK, who had extensive training in talking with and interacting with children of different ages and with complex communication needs. An essential part of the design was meeting the children in their homes, which made it possible to interact with the children and enable a deeper contextual understanding. To account for the children’s diverse cognitive and communicative abilities, a range of flexible data collection methods was employed, all aimed at capturing the child’s perspective. These methods included interviews, interactions, descriptions of non-verbal expressions, and an adapted photo elicitation method. The data collection, which included interviews for the comprehensive family study (ill child, siblings, and parents), took place over a duration of 4–7 h in each family on the same day.

#### Interviews

For children with verbal language, interviews were conducted to directly gather their views. Each interview was tailored to the child’s age and individual needs^44^ in collaboration with the parents before the interview day. According to the parents, the children had little knowledge or understanding of their increased risk of death, and therefore, the family’s words for the ill child’s condition—for example, “needing treatment” instead of “having cancer”—were used when talking with the ill child. The first author played or talked with the children ahead of the interview so that the child would feel safe enough to speak freely.^20,44^ Two children were interviewed alone, and two had one parent present for support. An open-minded approach was embraced, following the child’s cues to attain a deeper comprehension of their lifeworld and what mattered to them as they were asked to talk about the negative and positive aspects of being ill.

#### Interactions

An overall open attitude toward the child’s lifeworld was obtained. This approach involved spending time with the child to grasp what was important for them and gaining insights through interaction and contextual understanding. Due to the ill children’s differentiated understanding and communication skills, the interaction was adapted to each individual,^30^ such as interacting at the bedside, playing with the child in the living room or their rooms, or simply being nearby in the same room while family life happening around them. Field notes were written during and after the interaction with the ill child and structured within van Manen’s life existential of lived time, lived body, lived space, and lived relation.

#### Descriptions of non-verbal expressions

Siblings and parents provided descriptions of the ill child’s non-verbal expressions. Efforts were made to collect descriptions where the ill child’s own voice came forth through body language, sounds, reactions, actions, or other expressions. Seven siblings of five ill children, along with all parents, provided descriptions. The researcher talked with siblings individually and parents together, except for two parent couples who were interviewed separately in parts of the interview for practical reasons. The same approach used for interviews with the ill children was adopted for interviews with the younger siblings.

#### Adapted photo elicitation method

This approach was used to gain deeper insight into the ill child’s experiences,^45^ and the participants were asked to bring a picture or an object that could represent something important when living life with serious illness. Five of the children spontaneously showed objects within their rooms or homes during the interviews. For the remaining ill children, parents and siblings were questioned about what they believed the ill child would have brought to the interview if they had been able to do so. Two families had not prepared objects or pictures.

The interviews were digitally recorded and transcribed. All written material was de-identified, and all children were given fictive names. Only the main author knew the participants’ identities.

### Analysis

While the study is epistemologically and philosophically grounded in van Manen’s phenomenology of practice,^35^ we used Braun and Clarke’s six-phased reflexive thematic analysis to organize and enrich reflexivity in the analysis.^46,47^ Braun and Clarke’s six-phased method approach is theoretically flexible and can be applied within various frameworks.^46,47^ TBK conducted the analysis, while CC and AW read all the interviews/fieldnotes, engaged in discussions on analytical content, and provided intellectual contributions on drafts. A continuous focus in the analysis was being aware of the risk of overinterpreting data, given that some of the children lacked verbal language to express themselves. Capturing the child’s perspective was given priority throughout the analysis across all methods. Although this was more challenging in methods involving interactions and descriptions provided by siblings and parents, the intention was still to focus on the child’s perspective—whether through words or non-verbal expressions.

During the first phase, we read and re-read the interviews with the ill children, field notes, and interviews with parents and siblings to become thoroughly familiar with the content. Initial analytic insights were documented. In the second phase, the analytic question we asked was “What is it like to live with an LT/LL condition?” We inductively coded the data by generating labels that captured important features relevant to the research question (Table 3). We worked low-tech with codes on paper. Throughout this phase, special attention was given to distinguishing between what was expressed by the children and our interpretations.

**Table 3.** Analysis process.

Codes based on the analytic question, “What is it like to live with an LT/LL condition?”	Candidate themes	Themes
*Living with an LT/LL condition is to. . .* . . .miss being in kindergarten and being with friends. (Child 3, expressed by the ill child) . . .want to participate in family life. (Child 9, parents’ descriptions of situations where the ill child made sounds to get attention when not being involved with the family) . . .have focus on the activities in life. (Child 7, notes from interactions in which the ill child constantly focused on playing rather than issues concerning being ill)	Living through activity	Wanting to engage in life
. . .take medicine that makes you nauseous and tired. (Child 10, expressed by the ill child) . . .need shielding from sensory impressions. (Child 8, parent’s descriptions of situations where the ill child reacted to sounds and had to have shorter school days) . . .not being able to move the body independently because of spasticity and limited motor function. (Child 4, notes from interaction with the ill child)	Limitations of the body	
. . .feel bad when not being understood. (Child 1, parent’s descriptions of situations where the ill child reacted with anger and despair when new healthcare workers did not understand his language and his preferences). . . .be dependent on others to understand your nonverbal signals. (Child 4, notes from interaction with a child who relied on others to interpret his body language to determine when he was in pain)	Relational dependability	Being dependent on familiar relations
. . .like being with a sibling. (Child 6, a sibling’s and a mother’s description of situations where the ill child with reduced vision and motor function smiled and listened attentively when the sister entered the room) . . .take great pleasure in playing with a sibling. (Child 2, notes from interaction with the child, in which the child with reduced motor and cognitive abilities laughed and smiled during a special game on the floor with the sibling)	The special role of siblings	
. . .to enjoy the toys at the hospital. (Child 5, expressed by the ill child) . . .have a teddy bear as support in life. (Child 2, a sibling’s and parents’ descriptions of concrete situations where the ill child brought his teddy bear) . . .need well-known and cherished items to be able to be active, due to limited vision. (Child 11, notes from interaction with the ill child).	Things matter	The importance of cherished items

LT/LL, life-threatening or life-limiting.

In the third phase, we generated initial themes by examining the codes and collating them into candidate themes that had the same broader pattern of meaning. We aimed to develop themes that described the overarching phenomenon of living with an LT/LL condition across both cancer and genetic groups and children with and without verbal language. In this phase, we integrated the use of van Manen’s life existential by reflecting on the material through the lens of the different existentials.^35^ In the fourth phase, we developed and reviewed themes through a continuous back-and-forth process, following the hermeneutic circle between the parts and the whole. Codes were moved between the themes, and some themes were combined.

In the fifth phase, we delved deeper into the meaning of each theme by naming them in a way that captured the content within them. It became evident that the way a theme was labeled had a significant impact on how the child’s voice emerged. Some labels reflected a more interpreted perspective, while others presented a more straightforward understanding from the child’s point of view. Given the study’s focus on exploring children’s perspectives, we opted for labels that highlighted the child’s viewpoint. Sixth and last, we began the process of weaving the analytic narrative together and writing it up. Recognizing the significance of writing and rewriting in van Manen’s phenomenology of practice,^35^ we acknowledged that this phase provided insights into the material that was expressed within each theme during the writing process. Quotes (with fictitious names) and examples were chosen to highlight the meaning of each theme and were written into the material.

### Ethics

This study rests on a core belief in children’s agency and competence^29^ and children’s rights to express their opinions and be listened to, as outlined in the United Nations Declaration of the Rights of the Child, Article 12.^48^ The study complies with the Declaration of Helsinki,^49^ the European Code of Conduct for Research Integrity,^50^ and the Ethical Research Involving Children (ERIC guidelines).^51^ Approval was obtained from the Regional Ethics Board (ref number 251284), the Norwegian Center for Research Data (ref number 289184), and the research ethics boards at each hospital. The family received age-adapted information (written and oral) before the interviews, and for the youngest children and siblings, a symbol board made on an iPad with the Twinkl app^52^ was used to support the information before and during the interview in a concrete and understandable way. Parents provided electronic consent on behalf of themselves, siblings younger than 16 years, and the ill children, as they were either younger than 16 years old or unable to consent due to cognitive limitations. Siblings who were 16 years or older provided their own consent. In addition to obtaining formal consent, the children’s ongoing agreement was consistently sought throughout the interviews, and the children were encouraged to say “stop” or to use a stop sign whenever they wanted.^24^ The Service for Sensitive Data (TSD) was used to collect and store sensitive data. The interviews were recorded directly to the TSD server via the “Diktafon app.”

The ill children and their siblings received a small surprise gift for participating, given after the interview, according to the ERIC guidelines.^51^ After the interviews, the ill children and siblings were encouraged to talk with their parents about any emotional issues. The researcher contacted the parents 1–2 weeks after the interviews, and the parents reported that none of the ill children or siblings had described or expressed negative reactions to the interviews.

### Preunderstanding

The researchers’ preunderstanding was based on our professional experience in nursing, occupational therapy, and clinical medicine at different levels in the healthcare system. We have worked with various diagnoses, phases of illness, and qualitative research within PPC. The research group consisted of women who were also mothers.

## Results

**Figure 1.**
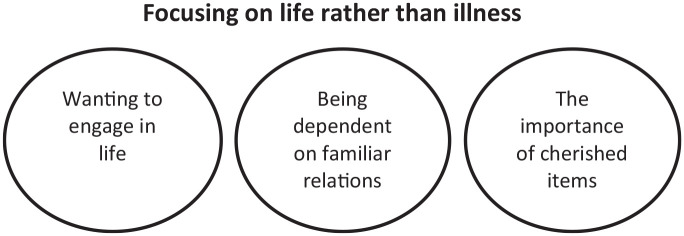
Results.

The findings showed that the children’s attention revolved around life rather than illness. Three themes were revealed: wanting to engage in life, being dependent on familiar relations, and the importance of cherished items (Figure 1).

### Wanting to engage in life

The children’s expressions of what mattered to them revolved around a focus on life rather than on illness. Overall, the children were occupied with playing and activities here and now. Some children who had to stay at home or in hospital during treatment or periods of infection risk conveyed their wishes to meet friends, play, or attend kindergarten and school, as expressed by a 7-year-old girl (Family 10): “It is so stupid that I have to miss school just to go to the hospital. I really want to play with my friends. And I love the things we do in school too.” For a 5-year-old girl (Family 3), the importance of friends and her desire to participate in life was supported by multiple data sources. During the interaction, the girl frequently returned to the topic of kindergarten, expressing her hope of soon being healthy enough to attend. She specifically focused on a picture of the children at the kindergarten hanging on the wall, repeatedly pointing at the picture and telling all of her friends’ names. Additionally, a description provided by her mother gave further insight: every morning, the girl would stand by the window, waving to the other children as they arrived or left for kindergarten, unable to join them due to the infection risk posed by her illness. The girl herself articulated the isolating nature of this experience, saying, “Actually, there is a really bad thing. My friends can’t come and visit me because they have the flu. Jan has the flu too, so I can’t visit him either.”

It was also possible to gain insight into the desire of children with limited verbal communication to participate in activities and family life, despite their complex medical and developmental conditions. Their participation in sensory experiences, holding hands with friends, listening to music, sitting on a parent’s lap, being with animals, or reading a book, was revealed as meaningful, highlighting the significance of these activities in their lives. This was evident in interaction with the children and in concrete descriptions of the children’s sounds and body language, such as smiling, laughter, and physical expressions, or conversely, calmness in their bodies during activities when they thrived and had a good time.

The meaning of participation was illustrated by the father of a 3-year-old boy (Family 4), who, when asked what his son might have brought to the interview if he could, suggested: “Maybe he would have brought an activity like his slide. That describes him well. Or all the thousand pictures of him being swung in a blanket. When we lie him down on the floor, his eyes are so big and his smile so broad.” This description was further supported during an interaction involving play with an indoor slide with the boy, his mother, and his two siblings. Though the ill boy needed significant help due to his severely limited motor function, he appeared happy and content, in the middle of family life. Similarly, interactions with an 8-year-old girl (Family 7) with limited verbal communication further highlighted the children’s wish to focus on other things than illness. While playing with Barbie dolls in her room, she consistently steered the conversation away from discussing her illness or hospital visits. Although her mother later mentioned that the girl had limited memory and attention span, it was interpreted that the girl deliberately avoided health-related topics to concentrate on the present moment and activities she enjoyed.

Through contextual insight, it was possible to understand that the children’s ability to participate in activities depended on a range of factors, such as the deterioration of their conditions or the necessity of “must-do” tasks like medication, mechanical ventilation management, and various medical procedures. These tasks could consume many hours per day, leaving little time and energy for childhood activities. Although this was not pointed out by the children themselves, it provided a deeper understanding of the challenges the children faced in participating in life in the way they had expressed a desire to do. Cognitive and motor disabilities significantly impacted some children’s ability to participate in various activities as well as limitations to sensory exposure, which could trigger epileptic seizures or general uneasiness.

### Being dependent on familiar relations

In various ways, the children expressed that they needed familiar relations to be understood, attain a sense of predictability, and unlock possibilities for play and participation. This was particularly evident for children expressing themselves in ways that were challenging to understand for those unfamiliar with the child. Through a description provided by the mother of a 17-year-old boy (Family 1), it was possible to understand the boy’s frustration when not being understood: “When there are new persons in the homeward that do not understand him, he gets really upset and angry. He yells loudly and tries to hit them. Once he broke his glasses.” Through contextual insight, it was further revealed that the need for familiar relations was not only due to communication needs but intimate knowledge of how the child had to be bodily positioned to participate in the best way despite their motor problems, reduced vision or hearing, or how the child liked to play. For example, interaction with a 5-year-old boy with severe vision impairment and significant spasticity revealed that he was routinely placed by his siblings and parents in positions that allowed him to participate as fully as possible.

The importance of familiar relations with siblings was particularly notable, as the ill children with verbal limitations often displayed heightened excitement when interacting with their brothers and sisters. In several descriptions of concrete situations provided by siblings or parents, it was possible to gain insight into how something “special” happened with the ill child’s body language when their siblings came into the room, or when the ill child heard the sibling’s voice. This was described by a 5-year-old sister (Family 6) as “He loves my voice.” Siblings often engaged in physical activities, such as playing together on the floor, which facilitated a unique connection with the ill child that transcended verbal communication. The sibling’s special role was that of a play companion—someone who could play with the child because they were a child themselves, while also being intimately familiar with the child’s needs and communication style. This was also demonstrated during interactions with a 5-year-old boy (Family 2) and his brother. When lifted from his chair to the floor where they played on a mat, the boy’s body language transformed—he smiled and laughed, and his eyes were wide and focused on his brother. A description provided by the boy’s mother supported this impression: “Even during times when Morten has been very ill, such as in the ambulance or on the respirator, he manages to smile whenever his brother arrives.”

### The importance of cherished items

Certain cherished items held significance for many children, indicating that these things “mattered” to them in various ways. The items fulfilled diverse needs, such as providing a sense of security, facilitating play, and fostering connections with the child.

In terms of security, many of the children possessed a teddy bear or a particular toy that had significant meaning in their lives, often given by a person with a special relationship to the child. These cherished items had been a companion throughout their life, accompanying them in hospitals, ambulance rides, examinations, holidays, and daily activities, offering comfort and support across a range of situations. For instance, a 4-year-old boy (Family 5) described his bond with his teddy bear Rifaz, stating, “This is my teddy bear Rifaz! It is a little blue dragon. He’s a bit worn out because I’ve tossed and tugged him. He is always with me. He always lies on the pillow with me.” Similarly, a 17-year-old boy (Family 11) without verbal language had a special relation to his teddy bear Ninni, an insight provided through a description from his mother: “We’d like to introduce you to Robert’s teddy bear, Ninni. Robert received Ninni from his grandmother when he was born, and ever since, Ninni has been by his side. Although Robert doesn’t play with Ninni, she remains the first item he packs, regardless of where he’s heading. Whether it’s a holiday trip or a visit to the hospital, Ninni is a must-bring companion.”

Other items served as a medium to play or interact with others. The brother of a 5-year-old boy (Family 2) without verbal language, described how a special toy served as a facilitator for play: “Sometimes me and Morten play with a toy parrot that makes weird sounds. Morten laughs when we play with it. He likes the sound and the colors. I can see that Morten is happy because he laughs and claps his hands. He does that when he is happy.” The children with verbal communication articulated that they found joy in items that were available in the hospital, not only because they facilitated play but also because they encouraged shared experiences with other children, for example, tricycles that allowed one child to sit in the back while someone else pedaled. A 4-year-old boy (Family 5) frequently expressed his positive feelings about the toys available at the hospital during the interview. When his mother suggested that his preference for one hospital over another was due to a negative medical experience at the latter, the boy strongly disagreed. He maintained that his dislike for the hospital was solely because it had no toys other than trains. This response was interpreted as an indication that the toys were significant from the child’s lifeworld and perspective.

Another aspect of the items was their ability to bridge connections and communication with the child. Stian’s parents (Family 1) described a situation during an extensive hospital stay where neither they nor the healthcare personnel could establish contact with him: “The nurses came in and made no contact with Stian. They asked us if we had any ideas about how to connect with him. We had seen an anatomically sized skeleton in a doctor’s office, so I said that we’d love to have that skeleton. He loves skeletons! So, we wheeled the skeleton over to his bed. And then he tried to say ‘great-grandmother’. I think that was the first time we’d heard his voice in a long time. Then he fell asleep again with the skeleton under his arm. But then we had achieved something.” The importance of skeletons was notable also during interaction with Stian, as numerous skeletons were hanging in his room.

## Discussion

The aim of this study was to explore the lived experiences of children living with LT/LL conditions. The study included children with and without verbal communication and provided insight into the lives of children seldom highlighted in research. The findings showed that the children’s attention revolved around life rather than their illness. Three themes that were important to the children were revealed: their desire to participate and engage in life, their need for familiar relations to be understood and enabled to participate, and the importance of special or cherished items in their lives.

The children in our study expressed a desire to engage in life, such as attending school, meeting friends, being involved in family activities, and engaging in enjoyable experiences despite their illness. Children with LT/LL conditions in other studies have also expressed their desire to undertake usual childhood activities,^12,53,54^ such as seeing friends, playing,^11^ taking part in their hobbies, going to school,^53^ or participating in other activities.^55^ Although many children experience burdensome symptoms from their condition, Namisango et al.^54^ showed that such symptoms become more of a concern when they affect the children’s ability to engage in daily activities.

Engaging in meaningful activities is associated with feelings of normality, which is an overarching desire for children.^9–12,53–57^ School is particularly important for their sense of normality, as it is an arena both for education and for maintaining friendships.^11^ For younger children, playing is important because it offers comfort as well as the development of cognitive, linguistic, and socio-emotional skills.^58,59^ Despite children’s right to participation and development,^48^ studies indicate that children living with LT/LL conditions experience deprivation in participation and play.^60–62^ Opportunities for play and engagement in meaningful activities can be overlooked or undervalued by parents and professionals, as the primary focus may be on alleviating the clinical symptoms of the illness and minimizing its psychological impact on the child.^60^ Factors such as frequent hospitalizations,^63^ infection precautions, the children’s health conditions, and limitations caused by social and physical environments can reduce children’s possibilities for participating in play and meaningful activities.^64^

Our study supports these findings, as isolation resulting from infection risks, the time-consuming nature of medical procedures, and sensory, motor, and cognitive disabilities frequently limited the children’s participation. Within this complex dynamic, there is a risk of overlooking children’s wishes for normality and participation in life.^18^ Holmen et al.’s^65^ review on patient-centered outcome measures (PROMs) found that central aspects of children’s lives, such as play, socializing, school participation, and autonomy, were not reflected in the identified PROMs.^65^ This highlights the importance of shifting focus from illness management alone to the inclusion of children’s desires for active engagement in life activities. By prioritizing meaningful participation and acknowledging children’s perspectives, healthcare professionals can optimize care outcomes for children living with LT/LL conditions. Meaningful activities can occur on different levels, including being present with where things happen, engaging in small, adapted activities in the child’s home, attending school, socializing, and participating in other enjoyable experiences. Practices aimed at supporting children’s agency and enabling them to express what is important to them can be implemented, for example, through the use of augmentative and alternative communication methods.^42^ This recognition should guide care plans and interventions.

Another finding in our study was the importance of familial relationships for the child to be understood, experience predictability, and be able to participate. In particular, children with cognitive and communicative disabilities relied heavily on family members and caregivers who understood their unique language, preferences, and optimal physical positioning to facilitate their participation in activities. This finding shows the importance of ensuring children have stable and familiar caregivers in their daily lives, who know them well and who understand their communication repertoire.

Our study also illustrated how parents, through their knowledge of the child, adapted the physical environment to facilitate their child’s engagement in activities and family life. Environmental factors can significantly impact play participation for children with LT/LL conditions,^62^ as physical surroundings can both limit and provide possibilities for participation and interaction with the world.^66^ However, balancing caregiving responsibilities with enabling the child to participate in activities such as school or socializing can be challenging for parents, as they face the difficult task of providing all the care and support required by the illness while striving to maintain a sense of normality in the child’s life.^67^

In addition, many activities may not be easily adaptable to accommodate the specific needs of children with LT/LL conditions, requiring significant effort from parents to modify or find suitable alternatives. Children with LT/LL conditions often rely on their parents or other adults for play due to isolation precautions and the need for assistance because of their complex conditions,^64,68–70^ and research shows that parents of children with complex disabilities may feel inadequate in engaging their children in meaningful activities.^69^ Children with LT/LL conditions, who may also have additional challenges such as epilepsy, hearing or visual impairments, cognitive impairments, or multiple complex conditions, often encounter difficulties interacting with their environment.^70^ In such circumstances, parents may require assistance to discover ways of engaging the child in activities customized to their specific abilities and preferences, especially if the child’s condition deteriorates and they are no longer able to participate in previously enjoyed activities. Parents can be helped to explore possibilities for their child’s engagement through adaptations to the physical surroundings and the selection of appropriate play materials, such as tactile exploration games and assistive technologies.^70^

A finding in our study was the unique role of siblings as significant familiar relationships for ill children. Several families expressed that “something special” happened with the ill child when they were with their siblings. Siblings demonstrated a unique ability to communicate and engage in play with ill children with communicative, cognitive, or motor disabilities, as they understood the child’s special needs while being children themselves, allowing for a different level of interaction and understanding. This finding underscores the importance of children being cared for at home as it highlights the valuable role of siblings in supporting and interacting with ill children within the familial environment. However, the holistic family perspective in PPC also includes being aware of siblings’ support needs, as siblings often take a considerable amount of responsibility and consideration when a family is living with an ill child.^39,71^

Further, we found that certain cherished items held significance for the children, providing a sense of security, facilitating play, and fostering connections. Research has shown that children miss their toys when hospitalized,^72^ highlighting the importance of understanding the role of such possessions in children’s lives without reducing it to adult perspectives or clichés.^73^ The lifeworld’s of children and adults are not the same, as the lifeworld of a child may exhibit different experiential qualities than that of an adult.^35^ In our study, the items served as existential constants in the child’s life, and in challenging situations such as hospital stays or medical procedures, having these familiar things nearby could offer a source of comfort and stability. Some items also fulfilled an important role as facilitators of play and therefore provided a sense of joy and normality in the children’s lives. This is in line with van Manen’s later work, in which he included “lived things” as an additional life existential and part of the human lifeworld.^74^ Van Manen^34^ drew upon the insights of Latour and Verbeek, who proposed that things possess agency just as human persons do. In this study, certain significant items in the children’s lives appeared to exert agencies, not only as projectors for activity and safety but also as life witnesses to the child’s journey and history. The clinical implications of the “importance of cherished items” highlight the need to recognize the emotional significance of certain objects and possessions in the lives of children with LT/LL conditions. Healthcare settings should accommodate the presence of such items and encourage their use as tools to enhance children’s well-being. By recognizing and honoring the child’s attachment to these things, caregivers can create meaningful interactions and foster a sense of trust and connection.

### Strengths and limitations

Several aspects must be considered to assess the trustworthiness of the study.^75,76^ The main credibility concern for this study stemmed from the use of multiple methods for data collection as well as several children having cognitive and communicative disabilities, along with young age. The use of multiple methods presents both strengths and limitations. On the one hand, employing a range of flexible methods was necessary to gain insight into the lived experiences of children with cognitive and communicative disabilities. On the other hand, the use of multiple methods introduces greater uncertainty regarding which data come from the various sources and whose voice is truly being represented. Questions may arise regarding whether it is truly possible to capture the child’s perspective when some of the methods rely on data collected through others. Therefore, we acknowledge the limitations inherent in the data collected through descriptions provided by others than the children themselves. It is important to recognize that although parents and siblings provided descriptions that aimed at capturing the child’s own perspective, the descriptions were chosen and filtered through the siblings and parents. Additionally, interpreting body language can be challenging and prone to misinterpretation, especially when children have cognitive limitations, which may lead to an over- or underestimation of the significance of their expressions. However, to ensure that children with cognitive and communicative disabilities are not excluded from research, it is essential to remain open to exploring methods that try to capture their voices.

To improve credibility, our primary focus across the multiple methods centered on comprehending what mattered to the ill child. The credibility of the study’s findings was enhanced by the researcher spending time in the homes of the families, facilitating a thorough understanding and deep insight into the context. Credibility may have been further enhanced by repeated visits to the children’s homes, but this was difficult due to the families’ time constraints and the long travel distances for the researcher.

Van Manen’s approach, which focuses on understanding the phenomenon through lived experiences drawn from concrete life episodes, proved well-suited for uncovering what was meaningful to the children, particularly the children who lacked verbal language or were younger. Initially, our focus was on children aged three and above. However, given that some of the initially recruited children had complex developmental disabilities, which prompted us to include the use of non-verbal expressions, we decided to include a 1-year-old child as the final participant and applied the same approach. Despite the varying ages, several of the children exhibited limited cognitive abilities, suggesting that their cognitive age was more closely aligned with each other than their chronological age.

The dependability of the research was heightened through the methodological consistency maintained throughout the study, with the same person conducting all interviews and transcriptions. Additionally, we adopted Braun and Clarke’s reflexive thematic analysis approach to enhance reflexivity in our analysis, complementing van Manen’s more open approach to thematic analysis. We found no contradictions between these two approaches; rather, van Manen’s phenomenological approach served as the foundation for philosophical, ontological, and methodological perspectives, while Braun and Clarke’s reflexive analysis gave structure to the descriptions of the analytical process.

To enhance confirmability, the author group consistently reflected on how their preunderstandings might impact the research process. The manuscript was read and commented on several times by all authors. Regarding transferability, which concerns the applicability of findings to different settings,^76^ descriptions of the context, participants, and analysis were provided, enabling readers to determine the relevance of the findings to their own settings. Despite the specific diagnoses of cancer and genetic conditions among the children in this study, the findings may hold relevance for various diagnoses, as our study centered on the broader phenomenon of living with an LT/LL condition.

## Conclusion

Above all, the study emphasizes that children with LT/LL conditions are children first and foremost, with a fundamental need to participate in meaningful activity, just like any other child. Alongside medical treatment and symptom management, professionals across various levels of PPC should acknowledge and prioritize children’s desire to engage in life. Practices should be implemented to support children’s agencies and expressions of what is important to them. This recognition should guide care plans and interventions at all levels of PPC.

## Supplemental Material

sj-docx-1-pcr-10.1177_26323524241301431 – Supplemental material for Focusing on life rather than illness: the lived experience of children with life-threatening and life-limiting conditions—a qualitative study Supplemental material, sj-docx-1-pcr-10.1177_26323524241301431 for Focusing on life rather than illness: the lived experience of children with life-threatening and life-limiting conditions—a qualitative study by Trine Brun Kittelsen, Charlotte Castor, Anja Lee, Lisbeth Gravdal Kvarme and Anette Winger in Palliative Care and Social Practice

## Declarations

**Ethics approval and consent to participate:** Ethical approval was obtained from the Regional Committees for Medical and Health Research Ethics in Norway (reference number 251284), the Norwegian Center for Research Data (reference number 289184), and the research ethics boards at each hospital involved in recruitment. Informed consent was obtained from all participants. Sensitive data were collected and stored using the Service for Sensitive Data (TSD). Interviews were recorded directly onto the TSD server via the Diktafon app.

**Consent for publication:** Informed consent for publication was provided by participants.

**Availability of data and materials:** The data is not publicly available. Due to the purpose limitation in the study approval from the Norwegian Center for Research Data (reference number 289184, art. 5.1 b), we are not allowed to share the transcribed material as individual privacy can be compromised.

## References

[1] Connor SR Downing J Marston J . Estimating the global need for palliative care for children: a cross-sectional analysis. J Pain Symptom Manage 2016; 53(2): 171–177. 27765706 10.1016/j.jpainsymman.2016.08.020

[2] Fraser LK Gibson-Smith D Jarvis S , et al. Estimating the current and future prevalence of life-limiting conditions in children in England. Palliat Med 2021; 35(9): 1641–1651. 33323043 10.1177/0269216320975308 PMC8532217

[3] World Health Organization. Planning and implementing palliative care services: a guide for program managers. Switzerland: World Health Organization, 2016.

[4] Arias-Casais NGE Rhee JY , et al. EAPC Atlas of Palliative Care in Europe 2019. Vilvoorde: EAPC Press, 2019.

[5] Fraser LK Lidstone V Miller M , et al. Patterns of diagnoses among children and young adults with life-limiting conditions: a secondary analysis of a national dataset. Palliat Med 2014; 28(6): 513–520. 24699788 10.1177/0269216314528743

[6] Levine DR Cuviello A Nelson C , et al. Hope-colored glasses: perceptions of prognosis among pediatric oncology patients and their parents. JCO Oncol Pract 2021; 17(6): e730–e739. 10.1200/OP.20.00762 33661701

[7] Fraser LK Parslow R . Children with life-limiting conditions in paediatric intensive care units: a national cohort, data linkage study. Arch Dis Child 2018; 103(6): 540–547. 28705790 10.1136/archdischild-2017-312638 PMC5965357

[8] Winger A Kvarme LG Løyland B , et al. Family experiences with palliative care for children at home: a systematic literature review. BMC Palliat Care 2020; 19(1): 1–165. 33099303 10.1186/s12904-020-00672-4 PMC7585197

[9] Comas Carbonell E Mateo-Ortega D Busquets-Alibés E . The psychological experience of pediatric oncology patients facing life-threatening situations: a systematic review with narrative synthesis. Palliat Support Care 2021; 19(6): 733–743. 33750507 10.1017/S1478951521000031

[10] McPoland P Grossoehme DH Sheehan DC , et al. Children’s understanding of dying and death: a multinational grounded theory study. Palliat Support Care 2024; 22: 213–220. 36960605 10.1017/S1478951523000287

[11] Coombes L Braybrook D Roach A , et al. Achieving child-centred care for children and young people with life-limiting and life-threatening conditions: a qualitative interview study. Eur J Pediatr 2022; 181(10): 3739–3752. 35953678 10.1007/s00431-022-04566-w PMC9371630

[12] Namisango E Bristowe K Murtagh FEM , et al. Towards person-centred quality care for children with life-limiting and life-threatening illness: self-reported symptoms, concerns and priority outcomes from a multi-country qualitative study. Palliat Med 2020; 34(3): 319–335. 32081084 10.1177/0269216319900137

[13] Cheng L Zhao X Ge Y , et al. The experiences of Chinese children 5- to 7-year-old during cancer treatment reflected through interviews and drawings. J Pediatr Hematol Oncol Nurs 2022; 39(2): 88–98. 34533397 10.1177/10434542211041919

[14] Jennifer WM Molly M Janice SW , et al. Agreement between child self-report and caregiver-proxy report for symptoms and functioning of children undergoing cancer treatment. JAMA Pediatr 2020; 174(11): e202861. 10.1001/jamapediatrics.2020.2861 PMC7445628 32832975

[15] Toro-Pérez D Limonero JT Guillen M , et al. Evaluating quality of life in pediatric palliative care: a cross-sectional analysis of children’s and parents’ perspectives. Eur J Pediatr 2024; 183: 1305–1314. 38112799 10.1007/s00431-023-05330-4 PMC10951001

[16] Friedel M Aujoulat I Brichard B , et al. The quality of life of children facing life-limiting conditions and that of their parents in Belgium: a cross-sectional study. Children (Basel) 2023; 10(7): 1167. 37508664 10.3390/children10071167 PMC10378398

[17] Clavering EK McLaughlin J . Children’s participation in health research: from objects to agents?: children’s participation in health research. Child Care Health Dev 2010; 36(5): 603611. 10.1111/j.1365-2214.2010.01094.x 20533922

[18] Ekberg K Ekberg S Weinglass L , et al. Attending to child agency in paediatric palliative care consultations: adults’ use of tag questions directed to the child. Sociol Health Illn 2022; 44(3): 566–585. 35089602 10.1111/1467-9566.13437 PMC9304193

[19] Kutrovátz K . Conducting qualitative interviews with children—methodological and ethical challenges. Corvinus J Soc Social Policy 2017; 8(2): 65–88.

[20] Kirk S . Methodological and ethical issues in conducting qualitative research with children and young people: a literature review. Int J Nurs Stud 2007; 44(7): 1250–1260. 17027985 10.1016/j.ijnurstu.2006.08.015

[21] Rabiee P Sloper P Beresford B . Doing research with children and young people who do not use speech for communication. Child Soc 2005; 19(5): 385–396. 10.1111/j.1365-2524.2005.00578.x 16048536

[22] Feudtner C Rosenberg AR Boss RD , et al. Challenges and priorities for pediatric palliative care research in the U.S. and similar practice settings: report from a Pediatric Palliative Care Research Network workshop. J Pain Symptom Manage 2019; 58(5): 909–917.e3. 10.1016/j.jpainsymman.2019.08.011 PMC8499153 31445136

[23] Tisdall EKM . The challenge and challenging of childhood studies? Learning from disability studies and research with disabled children. Child Soc 2012; 26(3): 181–191.

[24] Oulton K Gibson F Sell D , et al. Assent for children’s participation in research: why it matters and making it meaningful. Child Care Health Dev 2016; 42(4): 588–597. 27133591 10.1111/cch.12344

[25] Friedel M Aujoulat I Dubois A-C , et al. Instruments to measure outcomes in pediatric palliative care: a systematic review. Pediatrics 2019; 143(1): e20182379. 10.1542/peds.2018-2379 30530504

[26] Pimlott-Wilson H . Visualising children’s participation in research: Lego Duplo, rainbows and clouds and moodboards. Int J Soc Res Method 2012; 15(2): 135–148.

[27] Von Tetzchner S Basil C . Terminology and notation in written representations of conversations with augmentative and alternative communication. Augment Altern Commun 2011; 27(3): 141–149. 22008027 10.3109/07434618.2011.610356

[28] Bryan G Bluebond-Langner M Kelly D , et al. Studying children’s experiences in interactions with clinicians: identifying methods fit for purpose. Qual Health Res 2019; 29(3): 393–403. 30270755 10.1177/1049732318801358

[29] Montreuil M Carnevale FA . Participatory hermeneutic ethnography: a methodological framework for health ethics research with children. Qual Health Res 2018; 28(7): 1135–1144. 29542396 10.1177/1049732318757489

[30] Nilsson S Björkman B Almqvist A-L , et al. Children’s voices—differentiating a child perspective from a child’s perspective. Dev Neurorehabil 2015; 18(3): 162–168. 23924164 10.3109/17518423.2013.801529

[31] Punch S . Research with children: the same or different from research with adults? Childhood 2002; 9(3): 321–341.

[32] Maes B Nijs S Vandesande S , et al. Looking back, looking forward: methodological challenges and future directions in research on persons with profound intellectual and multiple disabilities. J Appl Res Intellect Disabil 2021; 34(1): 250–262. 33073444 10.1111/jar.12803

[33] Facca D Gladstone B Teachman G . Working the limits of “giving voice” to children: a critical conceptual review. Int J Qual Method 2020; 19: 160940692093339.

[34] van Manen M . Phenomenology of practice: meaning-giving methods in phenomenological research and writing. London: Routledge, 2016.

[35] van Manen M . Researching lived experience: human science for an action sensitive pedagogy. 2nd ed. London, ON: Althouse Press, 1997.

[36] Söderbäck M Coyne I Harder M . The importance of including both a child perspective and the child’s perspective within health care settings to provide truly child-centred care. J Child Health Care 2011; 15(2): 99–106. 21685225 10.1177/1367493510397624

[37] Sommer D Pramling Samuelsson I Hundeide K . Child perspectives and children’s perspectives in theory and practice. Dordrecht: Springer, 2010.

[38] Tong A Sainsbury P Craig J . Consolidated criteria for reporting qualitative research (COREQ): a 32-item checklist for interviews and focus groups. Int J Qual Health Care 2007; 19(6): 349–357. 17872937 10.1093/intqhc/mzm042

[39] Kittelsen TB Castor C Lee A , et al. “What about me?”: lived experiences of siblings living with a brother or sister with a life-threatening or life-limiting condition. Int J Qual Stud Health Well-being 2024; 19(1): 2321645. 38404038 10.1080/17482631.2024.2321645 PMC10898268

[40] Kittelsen TB Lorentsen VB Castor C , et al. It’s about living a normal life: parents’ quality of life when their child has a life-threatening or life-limiting condition: a qualitative study. BMC Palliat Care 2024; 23(1): 92. 38589835 10.1186/s12904-024-01417-3 PMC11003040

[41] CHIP. CHIP – Children in palliative care: OsloMet, https://uni.oslomet.no/chip/ (2023, accessed 01 June 2024).

[42] Thunberg G Johnson E Bornman J , et al. Being heard—supporting person-centred communication in paediatric care using augmentative and alternative communication as universal design: a position paper. Nurs Inq 2022; 29(2): e12426. 10.1111/nin.12426 34076320

[43] Cook AM Polgar JM Encarnacao P . Assistive technologies: principles and practice. 5th ed. St. Louis: Mosby, 2019.

[44] Kortesluoma R-L Hentinen M Nikkonen M . Conducting a qualitative child interview: methodological considerations. J Adv Nurs 2003; 42(5): 434–441. 12752864 10.1046/j.1365-2648.2003.02643.x

[45] Pain H . A literature review to evaluate the choice and use of visual methods. Int J Qual Method 2012; 11(4): 303–319.

[46] Braun V Clarke V. Thematic analysis: a practical guide. Los Angeles, CA: Sage, 2022.

[47] Braun V Clarke V . Supporting best practice in reflexive thematic analysis reporting in *Palliative Medicine*: a review of published research and introduction to the *Reflexive Thematic Analysis Reporting Guidelines* (RTARG). Palliat Med 2024; 38: 608–616. 38469804 10.1177/02692163241234800 PMC11157981

[48] UNICEF. Convention on the rights of the child: UNICEF, https://www.unicef.org/child-rights-convention/convention-text?page_router=true# (2022, accessed 08 May 2024).

[49] World Medical Association. World Medical Association Declaration of Helsinki: ethical principles for medical research involving human subjects. JAMA 2013; 310(20): 2191–2194. 24141714 10.1001/jama.2013.281053

[50] ALLEA. The European Code of Conduct for Research Integrity. Rev ed. Berlin: Berlin, 2023.

[51] Graham A Powell M Taylor N , et al. Ethical research involving children. Florence: UNICEF Office of research - Innocenti, 2013.

[52] Twinkl. Twinkl symbols, https://www.twinkl.no/symbols (2023, accessed 22 April 2022).

[53] Scott HM Coombes L Braybrook D , et al. Spiritual, religious, and existential concerns of children and young people with life-limiting and life-threatening conditions: a qualitative interview study. Palliat Med 2023; 37(6): 856–865. 36978266 10.1177/02692163231165101 PMC10227090

[54] Namisango E Bristowe K Allsop MJ , et al. Symptoms and concerns among children and young people with life-limiting and life-threatening conditions: a systematic review highlighting meaningful health outcomes. Patient 2019; 12(1): 15–55. 30361884 10.1007/s40271-018-0333-5

[55] Mitchell S Bennett K Morris A , et al. Achieving beneficial outcomes for children with life-limiting and life-threatening conditions receiving palliative care and their families: a realist review. Palliat Med 2020; 34(3): 387–402. 31431129 10.1177/0269216319870647 PMC7074600

[56] Ciobanu E Preston N . Hearing the voices of children diagnosed with a life-threatening or life-limiting illness and their parents’ accounts in a palliative care setting: a qualitative study. Palliat Med 2021; 35(5): 886–892. 33765880 10.1177/02692163211000238

[57] Ribbers S Wager J Hartenstein-Pinter A , et al. Core outcome domains of pediatric palliative care for children with severe neurological impairment and their families: a qualitative interview study. Palliat Med 2020; 34(3): 309–318. 31680627 10.1177/0269216319885818

[58] Jones M . The necessity of play for children in health care. (The children’s corner: perspectives on supportive care). Pediatr Nurs 2018; 44(6): 303.

[59] Verstraete J Ramma L Jelsma J . Item generation for a proxy health related quality of life measure in very young children. Health Qual Life Outcomes 2020; 18(1): 11. 31937311 10.1186/s12955-020-1271-1 PMC6961344

[60] Boucher S Downing J Shemilt R . The role of play in children’s palliative care. Children (Basel) 2014; 1(3): 302–317. 27417481 10.3390/children1030302 PMC4928732

[61] Jasem ZA Lambrick D Randall DC , et al. The social and physical environmental factors associated with the play of children living with life threatening/limiting conditions: a Q methodology study. Child Care Health Dev 2022; 48(2): 336–346. 34806192 10.1111/cch.12933

[62] Jasem ZA Darlington A-S Lambrick D , et al. Play in children with life-threatening and life-limiting conditions: a scoping review. Am J Occup Ther 2020; 74(1): 7401205040p1–7401205040p14. 10.5014/ajot.2020.033456 PMC7018458 32078515

[63] Ebrahimpour F Mirlashari J Hosseini ASS , et al. Symbols of hope on pediatric oncology ward: children’s perspective using photovoice. J Pediatr Oncol Nurs 2021; 38(6): 385–398. 34541954 10.1177/10434542211041934

[64] Jasem ZA Randall DC Darlington A-S , et al. Caregivers’ perspectives on the social and physical environmental factors associated with the play of their children with palliative care needs: a Q methodology study. J Child Health Care 2023; 27(1): 91–104. 35275767 10.1177/13674935211044875

[65] Holmen H Winger A Steindal SA , et al. Patient-reported outcome measures in children, adolescents, and young adults with palliative care needs: a scoping review. BMC Palliat Care 2023; 22(1): 1–148. 37798706 10.1186/s12904-023-01271-9 PMC10557323

[66] Taylor RR Kielhofner G . Kielhofner’s model of human occupation: theory and application. 5th ed. Philadelphia, PA: Wolters Kluwer, 2017.

[67] Sourkes B Frankel L Brown M , et al. Food, toys, and love: pediatric palliative care. Curr Probl Pediatr Adolesc Health Care 2005; 35(9): 350–386. 16301200 10.1016/j.cppeds.2005.09.002

[68] Jasem ZA Darlington A-S Lambrick D , et al. ‘Eat, sleep, internet and talk’: an exploratory study of play profile for children living with palliative care needs. Palliat Care Social Pract 2022; 16: 26323524221105100. 10.1177/26323524221105100 PMC9260576 35811780

[69] Brodin J . Diversity of aspects on play in children with profound multiple disabilities. Early Child Dev Care 2005; 175(7–8): 635–646.

[70] Caprino F Stucci V . Play in children with multiple disabilities. Warsaw, Poland: De Gruyter Open, 2017, pp. 147–154.

[71] Tay J Widger K Stremler R . Self-reported experiences of siblings of children with life-threatening conditions: a scoping review. J Child Health Care 2022; 26(4): 517–530. 34116616 10.1177/13674935211026113 PMC9667075

[72] Nabors L Liddle M . Perceptions of hospitalization by children with chronic illnesses and siblings. J Child Fam Stud 2017; 26(6): 1681–1691.

[73] Langeveld MJ . How does the child experience the world of things? Phenomenol Pedagogy 1984; 2: 215–223.

[74] Manen MV . Phenomenology of practice. Phenomenol Pract 2007; 1(1): 11–30.

[75] Lincoln YS Guba EG . Naturalistic inquiry. Beverly Hills, CA: Sage, 1985.

[76] Connelly LM . Trustworthiness in qualitative research. Medsurg Nurs 2016; 25(6): 435–436. 30304614

