# Supplementary material for: Focusing on life rather than illness: the lived experience of children with life-threatening and life-limiting conditions—a qualitative study
Source: Palliat Care Soc Pract. 2024 Nov 29;18:26323524241301431. doi: 10.1177/26323524241301431 (PMC11607763; doi:10.1177/26323524241301431)
Supplement: sj-docx-1-pcr-10.1177_26323524241301431 – Supplemental material for Focusing on life rather than illness: the lived experience of children with life-threatening and life-limiting conditions—a qualitative study [file sj-docx-1-pcr-10.1177_26323524241301431.docx]

**Consolidated criteria for reporting qualitative studies (COREQ): 32 item checklist**

Tong A, Sainsbury P, Craig J. Consolidated criteria for reporting qualitative research (COREQ): a 32-item checklist for interviews and focus groups. Int J Qual Health Care. 2007;19(6):349-357.

| No | Item | Guide questions/description | Mentioned in manuscript (Y/N) | Location in manuscript |
| --- | --- | --- | --- | --- |

**Domain 1: Research team and reflexivity**

| Personal Characteristics | | | | |
| --- | --- | --- | --- | --- |
| 1 | Interviewer/facilitator | Which author/s conducted the interview or focus group? | Yes | Data collection section |
| 2 | Credentials | What were the researcher’s credentials? E.g. PhD, MD | Yes | Author contributions |
| 3 | Occupation | What was their occupation at the time of the study? | Yes | Preunderstanding |
| 4 | Gender | Was the researcher male or female? | Yes | Preunderstanding |
| 5 | Experience/training | What experience or training did the researcher have? | Yes | Preunderstanding |
| Relationship with participants | | | | |
| 6 | Relationship established | Was a relationship established prior to study commencement? | Yes | Recruitment and participants |
| 7 | Participant knowledge of the interviewer | What did the participants know about the researcher? e.g. personal goals, reasons for doing the research | No | Comment: The children were informed that the researcher aimed to "understand what it is like to live with an illness." |
| 8 | Interviewer characteristics | What characteristics were reported about the interviewer/facilitator? e.g. Bias, assumptions, reasons and interests in the research topic | Yes | Ethics |

**Domain 2: study design**

| Theoretical framework | | | | |
| --- | --- | --- | --- | --- |
| 9 | Methodological orientation and Theory | What methodological orientation was stated to underpin the study? e.g. grounded theory, discourse analysis, ethnography, phenomenology, content analysis | Yes | Design |
| Participant selection | | | | |
| 10 | Sampling | How were participants selected? e.g. purposive, convenience, consecutive, snowball | Yes | Recruitment and participants |
| 11 | How were participants selected? e.g. purposive, convenience, consecutive, snowball | How were participants approached? e.g. face-to-face, telephone, mail, email | Yes | Recruitment and participants |
| 12 | Sample size | How many participants were in the study? | Yes | Recruitment and participants |
| 13 | Non-participation | How many people refused to participate or dropped out? Reasons? | Yes | Recruitment and participants |
| Setting | | | | |
| 14 | Setting of data collection | Where was the data collected? e.g. home, clinic, workplace | Yes | Datacollection |
| 15 | Presence of non-participants | Was anyone else present besides the participants and researchers? | Yes | Datacollection |
| 16 | Description of sample | What are the important characteristics of the sample? e.g. demographic data, date | Yes | Recruitment and participants |
| Data collection | | | | |
| 17 | Interview guide | Were questions, prompts, guides provided by the authors? Was it pilot tested? | Yes | Datacollection |
| 18 | Repeat interviews | Were repeat interviews carried out? If yes, how many? | No | Comment: Repeated interviews were not carried out due to the families' busy schedules and the travel distance for the researcher, and therefore are not mentioned in the manuscript. |
| 19 | Audio/visual recording | Did the research use audio or visual recording to collect the data? | Yes | Datacollection |
| 20 | Field notes | Were field notes made during and/or after the interview or focus group? | Yes | Datacollection |
| 21 | Duration | What was the duration of the interviews or focus group? | Yes | Datacollection |
| 22 | Data saturation | Was data saturation discussed? | No | Discussed in development of the project plan, but not mentioned in the manuscript. |
| 23 | Transcripts returned | Were transcripts returned to participants for comment and/or correction? | No | Comment: Transcripts were not returned to participants for comments. |

**Domain 3: analysis and findings**

| Data analysis | | | | |
| --- | --- | --- | --- | --- |
| 24 | Number of data coders | How many data coders coded the data? | Yes | Analysis |
| 25 | Description of the coding tree | Did authors provide a description of the coding tree? | Yes | Analysis |
| 26 | Derivation of themes | Were themes identified in advance or derived from the data? | Yes | Analysis |
| 27 | Software | What software, if applicable, was used to manage the data? | Yes | Analysis |
| 28 | Participant checking | Did participants provide feedback on the findings? | No | Comment: Feedback was not provided on the findings. |
| Reporting | | | | |
| 29 | Quotations presented | Were participant quotations presented to illustrate the themes / findings? Was each quotation identified? e.g. participant number | Yes | Analysis/Results |
| 30 | Data and findings consistent | Was there consistency between the data presented and the findings? | Yes | Analysis/Results |
| 31 | Clarity of major themes | Were major themes clearly presented in the findings? | Yes | Analysis/Results |
| 32 | Clarity of minor themes | Is there a description of diverse cases or discussion of minor themes? | Yes | Results |
